# Akt Phosphorylation Influences Persistent Chlamydial Infection and *Chlamydia*-Induced Golgi Fragmentation Without Involving Rab14

**DOI:** 10.3389/fcimb.2021.675890

**Published:** 2021-06-08

**Authors:** Xiaobao Huang, Jinfeng Tan, Xiaohong Chen, Mingna Liu, Huiling Zhu, Wenjing Li, Zhenjian He, Jiande Han, Chunguang Ma

**Affiliations:** ^1^ Department of Dermatology, The First Affiliated Hospital, Sun Yat-sen University, Guangzhou, China; ^2^ Department of Gynecology, The First Affiliated Hospital, Sun Yat-sen University, Guangzhou, China; ^3^ Department of Dermatology, The First Affiliated Hospital of Guangzhou Medical College, Guangzhou, China; ^4^ School of Public Health, Sun Yat-sen University, Guangzhou, China

**Keywords:** Chlamydia trachomatis, acute infection, persistent infection, Rab14, Akt phosphorylation, Golgi fragmentation

## Abstract

*Chlamydia trachomatis* is an obligate intracellular bacterium that causes multiple diseases involving the eyes, gastrointestinal tract, and genitourinary system. Previous studies have identified that in acute chlamydial infection, *C. trachomatis* requires Akt pathway phosphorylation and Rab14-positive vesicles to transmit essential lipids from the Golgi apparatus in survival and replication. However, the roles that Akt phosphorylation and Rab14 play in persistent chlamydial infection remain unclear. Here, we discovered that the level of Akt phosphorylation was lower in persistent chlamydial infection, and positively correlated with the effect of activating the development of *Chlamydia* but did not change the infectivity and 16s rRNA gene expression. Rab14 was found to exert a limited effect on persistent infection. Akt phosphorylation might regulate *Chlamydia* development and *Chlamydia*-induced Golgi fragmentation in persistent infection without involving Rab14. Our results provide a new insight regarding the potential of synergistic repressive effects of an Akt inhibitor with antibiotics in the treatment of persistent chlamydial infection induced by penicillin.

## Introduction

*Chlamydia trachomatis* (*C. trachomatis*) is an obligate intracellular bacterium that can cause several diseases and sequelae in human beings. It has been reported that *Chlamydia* infection is the most common sexually transmitted disease in the US (CDC, 2019). Only in 2018, over one million cases were reported to CDC. The *Chlamydia* infections of the female genital tract are usually asymptomatic but can lead to cervicitis, pelvic inflammatory disease (PID), and severe complications such as ectopic pregnancy and tubal factor infertility (TFI) (Tsevat et al., 2017).

In host cells, *C. trachomatis* has a unique developmental cycle that is formed by two alternative forms, infectious non-replicative elementary bodies (EBs) and replicative, non-infectious reticulate bodies (RBs) (Abdelrahman and Belland, 2005; Stephens et al., 2011). The complex of bacteria and vesicles in which bacteria develop and replicate are called inclusions. When the normal life cycle is disturbed by a variety of stresses, such as amino acid deficiency (Beatty et al., 1994), nutrient depletion (Capmany and Damiani, 2010), antibiotics (Zhu et al., 2014; Xue et al., 2017), immunological factors, and interferon-gamma (IFN-γ) (Beatty et al., 1993), the inclusions become smaller and harbor aberrant reticulate bodies (ABs), with slow metabolism and weakened infectivity. This results in persistent chlamydial infection, which is thought to be associated with female infertility (Witkin et al., 2017). Persistent infections can be reactivated by removing the stressful conditions. However, due to the difficulty of diagnosis and resistance to antibiotics, persistent infection remains a public health problem (Patton et al., 1994).

*Chlamydia trachomatis* needs to acquire nutrients, including amino acids, nucleotides, and lipids, from the host cells (van Ooij et al., 2000; Moore et al., 2008; Saka and Valdivia, 2010; Mehlitz et al., 2017). Chlamydial inclusions intercept nutrients from trans-Golgi network (TGN)-derived vesicles (Carabeo et al., 2003; Moore et al., 2008), multivesicular bodies (MVBs) (Beatty, 2006; Robertson et al., 2009; Gambarte Tudela et al., 2015), and lipid droplets (Kumar et al., 2006). In eukaryotic cells, Rab GTPases control the vesicle transport. They exert their functions through the GTP/GDP cycle, shifting from a membrane-associated active state when GTP-bound to an inactive cytosolic GDP-bound state. Phosphatidylinositol-3-kinase (PI3K)/Akt/Akt Substrate of 160 kDa (AS160) signaling pathway has been demonstrated to regulate Rab GTP/GDP cycling (Klip et al., 2014). Akt phosphorylation triggers the inactivation of AS160, and consequently, slows GTP hydrolysis and prolongs the active duration of Rabs. *C. trachomatis* is capable to utilize the Akt/AS160 pathway to enhance the recruitment of Rab14-positive vesicles with Golgi-derived sphingolipids (Capmany et al., 2019). Reduction of Rab14 and the presence of Akt inhibitors both impair *Chlamydia* development and provoke the appearance of ABs (van Ooij et al., 2000; Capmany and Damiani, 2010).

In this study, we investigated how Akt phosphorylation and Rab14 affect *Chlamydia* development in persistent infection. We discovered that in persistent chlamydial infection, the level of Akt phosphorylation was lower and positively correlated with the effect of activating the development of *Chlamydia* but did not change the infectivity. However, Rab14 exerted a limited effect on persistent infection. Akt phosphorylation might regulate *Chlamydia*-induced Golgi fragmentation in persistent infection without involving Rab14.

## Materials and Methods

### Acute and Persistent Chlamydial Infection

HeLa cells were cultured in RPMI 1640 medium (Gibco, USA) supplemented with 10% fetal bovine serum (FBS) (Gibco, USA) at 37°C in 5% CO_2_. *C. trachomatis* serovar D at an MOI of 1 was added and incubated with the cells by centrifugation at 3400×*g* at 37°C for 1 hour. The samples were then incubated for another hour at 37°C in 5% CO_2_. Extracellular bacteria were removed by aspirating the supernatant followed by the addition of fresh medium supplemented with 10% FBS and 0.5% glucose, and the cells were maintained under the same conditions until they were harvested at different time points.

For persistent infection, the procedures were the same as mentioned above except for the addition of 100 U/ml penicillin (Lukang Pharmaceutical Co., Ltd., Shandong, China) to the medium after centrifugation (Skilton et al., 2009). Uninfected cells cultured in medium with/without 100U/ml penicillin were used as controls.

### Antibodies and Reagents

In this study, we used the following antibodies: goat polyclonal to *Chlamydia trachomatis* MOMP coupled to FITC (Abcam, ab30951, USA); rabbit polyclonal anti-RAB14 (Abcam, ab28639, USA); rabbit monoclonal anti-GM130 (Abcam, ab52649, USA); rabbit polyclonal anti- GOLGA5/Golgin-84 (Abcam, ab224040, USA); rabbit polyclonal anti-Akt (pan) (Cell Signaling, 4685, USA); rabbit polyclonal anti-phosphorylated Akt (Ser-473) (Cell Signaling, 4060, USA); rabbit polyclonal anti-AS160 (Affinity, AF7630, USA); rabbit polyclonal anti-phosphorylated AS160 (Ser-318) (Affinity, AF2317, USA); rabbit polyclonal anti-GAPDH (Cell Signaling, 2118, USA); goat anti-rabbit HRP-conjugated IgG (ABclonal, AS007, China), and goat anti-rabbit Cy3-labeled IgG (ABclonal, AS007, China). To activate Akt, cells were pretreated with SC79 (Beyotime, SF2730, China) for 30 min. To inhibit Akt, Akt Inhibitor VIII (iAkt) (Beyotime, SF2730, China) was added to the culture medium at the indicated post-infection (p.i.) time. Dimethyl sulfoxide (DMSO) (Sigma-Aldrich, France) was used as a control for SC79 and iAkt.

### Immunofluorescence

For immunofluorescence staining, cells were fixed with 4% paraformaldehyde, permeabilized in 0.5% (v/v) Triton X-100 (Sigma–Aldrich, USA), and then blocked with 1% (w/v) BSA (Thermo Scientific, USA). Cells were then incubated with the primary antibody for 1.5h, washed in PBS, and incubated with Cy3-coupled secondary antibodies. Finally, the samples were counterstained with DAPI (Beyotime, China). Immunofluorescence images were acquired using a fluorescence microscope (Olympus, Japan).

### Immunoblotting

Uninfected and infected HeLa cells were lysed in RIPA buffer (Thermo Scientific, USA) supplemented with protease and phosphatase inhibitors (KeyGEN, China) at the indicated time points, and the protein concentrations were determined using the Pierce BCA kit (Thermo Scientific, USA). Total proteins samples (15 g/lane) were separated on a reducing SDS-PAGE gel and then transferred onto a PVDF membrane. The membrane was incubated overnight with a primary antibody to detect the desired proteins. Protein loading was assessed using rabbit polyclonal anti-GAPDH (1:1000). The membranes were then washed and incubated with goat anti-rabbit HRP-conjugated IgG (1:5000) and developed using the Immobilon ECL Ultra Western HRP Substrate (Millipore, WBKLS0100, USA) in an ImageQuant LAS4000 (GE Healthcare Life Sciences, Japan).

### Quantitative PCR (qPCR)

Total RNA was extracted from infected HeLa cells with or without penicillin at the indicated time points using TRIzol reagent (Invitrogen, USA). The yield of RNA was determined using a NanoDrop 2000 spectrophotometer (Thermo Scientific, USA). Reverse transcription reactions were performed using *Evo M-MLV* RT Premix for qPCR (Accurate Biotechnology Co., Ltd., China) according to the manufacturer’s instructions. Quantitative PCR was performed with SYBR^®^ Green Premix *Pro* Taq HS qPCR Kit II (Accurate Biotechnology Co., Ltd., China) targeting the chlamydial 16s rRNA gene. PCR cycling conditions were as follows: initial denaturation for 2 min at 95°C, followed by 40 cycles of denaturation at 95°C for 30 s, annealing at 58°C for 30 s, and extension at 72°C for 30 s. Expression levels of the target gene were calculated using 2^−ΔΔCt^, relative to the reference gene (GAPDH), allowing for the calculation of fold change relative to the control. The primers for 16s rRNA used were (Eszik et al., 2016): forward: 5’-CACAAGCAG TGGAGCATGTGGTTT-3’, reverse: 5’-ACTAACGATAAGGGTTGCGCTCGT-3’.

### Infectivity Assay

Infected cells were lysed at 48h p.i. The cell lysate was centrifuged for 10 minutes at 500×*g* to remove cell debris, and diluted 10-fold. The lysates were titrated with fresh HeLa cells. After 48h, the number of inclusions formed by chlamydial progeny was assessed by microscopic analysis at ×200 magnification. The average number of inclusion forming units/mL (IFUs/mL) was calculated for each sample and experimental condition.

### Rab14 Silencing

HeLa cells were grown on either 6-well plates or 24-well plates until they reached 30-50% confluence, and transfected with siRNA-Rab14 or control-siRNA (100nM final concentration) (RiboBio, China) using riboFECT™ CP Reagent (RiboBio, China). At 72h post-transfection, cells were infected with *C. trachomatis*, as described above, and incubated for the indicated periods for further manipulation. We used immunoblotting to confirm the decrease in Rab14 protein expression at 72h post-transfection.

### Cell Viability Assay

Cell viability was assessed using the Cell Counting kit-8 (CCK-8) (Servicebio, G4103, China). Briefly, 5000 HeLa cells per well were seeded into 96well plates. The cells were treated with different chemicals and incubated for different times to evaluate the toxicity of the chemicals. CCK-8 reagent was added to the wells and incubated at 37°C for 2 h. Absorbance was measured at 450 nm using a microplate reader (BioTek 800TS, USA).

### Statistical Analysis

GraphPad Prism 9 (GraphPad Software, La Jolla, CA, USA) was used for graphing and data analysis was performed using SPSS 25.0 (SPSS Inc, Chicago, IL, USA). Quantitative data were presented as means ± standard deviation (SD). The quantitative data were tested for normality using the Shapiro-Wilk test. Kruskal-Wallis followed by Dunn’s multiple comparisons test was used for evaluating of inclusion area. One-way ANOVA with Bonferroni’s multiple comparisons test was used for evaluation of infectivity assay and CCK-8 assay. Differences were considered significant at *P<0.05, ** *P*<0.01, *** *P*<0.001, ****P<0.0001.

## Results

### Akt Phosphorylation Levels Exhibit a Dynamically Decreased Pattern in Persistent Chlamydial Infection

**Figure 1:**
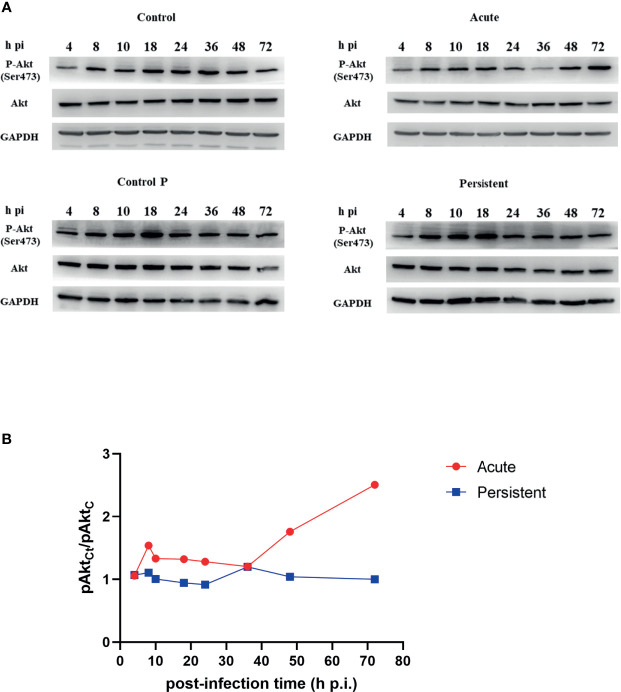
Different Akt phosphorylation level in acute and persistent chlamydial infection. **(A)** HeLa cells were infected with *C. trachomatis* (MOI 1) and cultured without/with penicillin. The lysates were harvested at the indicated periods of time. Uninfected cells treated without/with penicillin were used as control (Control and Control P). Cells were lysed in RIPA buffer with protease and phosphatase inhibitors and proteins were separated by SDS-PAGE. Proteins were transferred to PVDF membranes followed by immunoblotting with antibodies specific for Akt and phosphorylated Akt. GAPDH was used as a loading control. Cells were FBS starved 4 h before sampling. The results are representative of three independent experiments. **(B)** Relative phosphorylation level of Akt in chlamydial infected cells compared to pAkt in uninfected cells.

It has been described that Akt is phosphorylated during the entire chlamydial developmental cycle (Capmany et al., 2019). Here, we detected the phosphorylation level of Akt at different post-infection times of acutely or persistently infected cells using immunoblotting analysis. Due to the induction of Akt phosphorylation by a component of FBS (Watton and Downward, 1999), we first examined the level of Akt phosphorylation treated with FBS deprivation for different time periods, and the data are shown in **Figure S1**. Based on these results, we decided to perform the experiment after 4 h of FBS starvation. Phosphorylated Akt (pAkt) and total Akt expression levels in uninfected cells treated without/with penicillin remained steady. **Figure 1A** shows the typical immunoblotting results for the infected and uninfected cells. **Figure 1B** shows the ratio of pAkt in acutely or persistently infected cells, relative to uninfected cells, at different post-infection times based on three independent experiments. In acutely infected cells, phosphorylated Akt levels were elevated progressively since 36h p.i. Moreover, there were two peaks of phosphorylation in persistently infected cells: at the middle (8 h p.i.), and later stages (36 h p.i.) of the bacterial developmental cycle.

The level of pAkt in persistently infected cells was lower than that in acutely infected cells, at the same post-infection time except at 4h p.i. and 36h p.i. The dominance of pAkt in acutely infected cells was observed in the later stages of the developmental cycle (48h p.i. to 72h p.i.). Total Akt expression levels in two infection states remained unaltered with the time of infection. In addition, we detected the phosphorylation level of Akt Substrate of 160 kDa (AS160), and compared the level of phosphorylated AS160 (pAS160) between infected cells and uninfected cells, at different post-infection times respectively (**Figure S2**). pAS160 level were observed to vary in a time-dependent manner, similar to those of pAkt. Taken together, our findings revealed that Akt phosphorylation is dynamic during different stages of chlamydial infection, and the level had a robust decrease in persistent infection.

### The Recruitment Pattern of Rab14 in Acute and Persistent Chlamydial Infection

To determine whether there was a difference in Rab14 recruitment between persistent infection and acute infection during the developmental cycle, we detected the intracellular distribution of endogenous Rab14 in cells of two infection states, at different post-infection times. Uninfected cells cultured without/with penicillin were used as control groups (control and control P).

**Figure 2:**
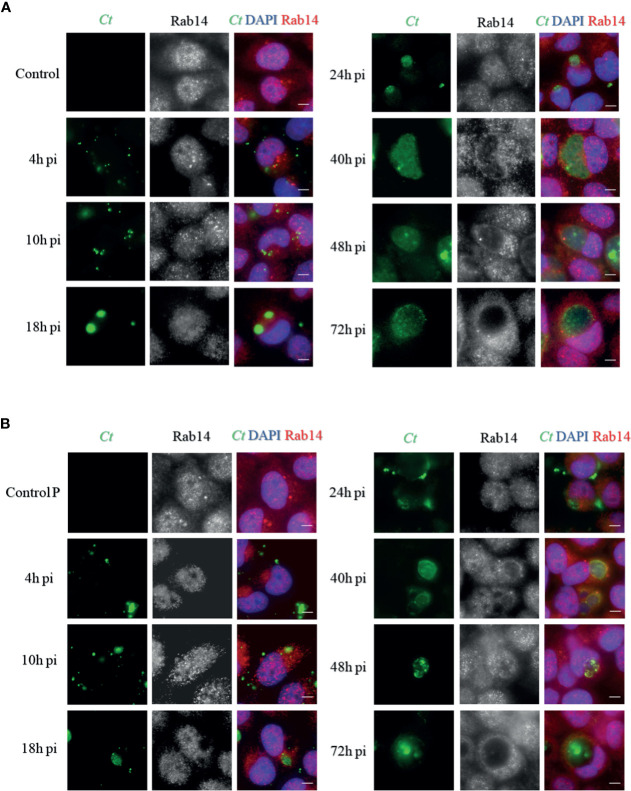
Distribution of Rab14 in acute and persistent chlamydial infection. **(A)** HeLa cells were infected with *C. trachomatis* serovar D (MOI 1) and fixed at indicated post-infection (p.i.) times. Uninfected cells were used as a control. **(B)** HeLa cells were infected with *C. trachomatis* serovar D (MOI 1) cultured with 100U/mL penicillin and fixed at indicated post-infection (p.i.) times. Uninfected cells cultured with 100U/mL penicillin were used as control. **(A, B)** *C. trachomatis* were stained with FITC-labeled anti-chlamydia MOMP (green). The Rab14 was stained with rabbit monoclonal anti-RAB14 antibody labeled with Cy3 (red). Bacterial and eukaryotic DNA was labeled with DAPI (blue). Scale bar = 20μm. Images are representative of two independent experiments.

Exposure to penicillin did not alter the Rab14 distribution in uninfected cells. However, although Rab14 presented dispersedly at the early stage (4h p.i.) of both infection states, Rab14 recruitment became associated to the inclusions and delimited the border of inclusions as the infection was processed. In the acute state, Rab14 tended to surround the inclusions since the 24h p.i. and gradually showed a rim-like staining pattern (**Figure 2A**). This indicates that Rab14 is closely associated with inclusions in acutely infected cells. In contrast, the chlamydial inclusions became smaller in the cells with persistent chlamydial infection induced by penicillin. In addition, the rim-like shape pattern of Rab14 was observed in the later stages of development (40h p.i to 72h p.i), but to a lesser extent (**Figure 2B**). However, our experiments might imply the potential pattern of Rab14 recruitment in acutely and persistently infected cells because we did not show the GTP-loading Rab14.

### Effect of Akt Phosphorylation on Inclusion Growth and Infectivity

As Akt phosphorylation is associated with chlamydial infection, we next aimed to confirm the effect of Akt phosphorylation on the bacterial development and infectivity in the presence of SC79, an Akt activator, and Akt Inhibitor VIII (iAkt). First, we confirmed the non-toxicity of SC79, and the dose-dependent toxicity of iAkt to HeLa cells by CCK-8 assay (**Figures S3A, B**). Next, we detected the effects of SC79 and iAkt on Akt phosphorylation by immunoblotting and found that they both exert dose-dependent effects (**Figures S3C, D**). Consequently, we used 4μg/mL SC79 or 5μM iAkt in subsequent experimental procedures. DMSO was used as the control.

**Figure 3:**
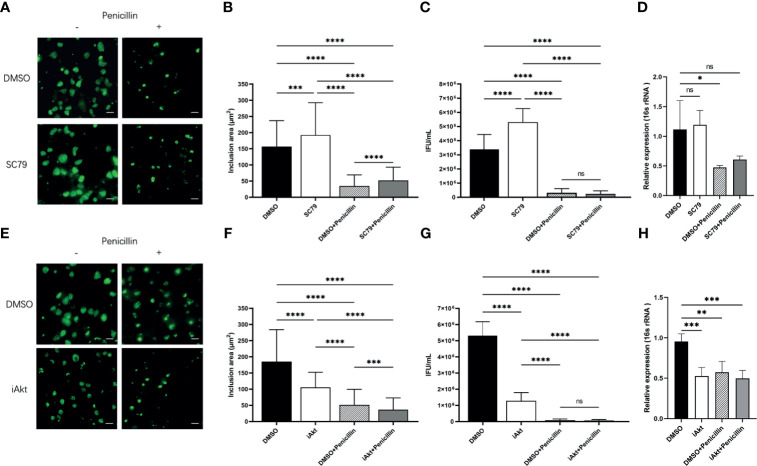
Effect of Akt phosphorylation on inclusion growth and infectivity. **(A)** HeLa cells were pretreated with either DMSO or 4μg/mL SC79 for 30min and then infected with *C. trachomatis* (MOI 1). Infected cells were cultured without/with penicillin and fixed at 48h p.i. *C. trachomatis* were stained with FITC-labeled anti-chlamydia MOMP (green). Inclusion area was measured by immunofluorescence microscopy. Scale bar = 50μm. **(B)** Quantification of chlamydial inclusion area demonstrated in **(A)**. **(C)** Infected HeLa cells were lysed at 48h p.i. The infectious particles (EBs) released were titrated on fresh HeLa cells by counting the Inclusion Forming Units (IFU) 48 h later as described in *Material and Methods*. **(D)** The total RNA was extracted from the infected cells at 48h p.i. and then the expression of 16s rRNA gene was tested by qPCR. **(E)** HeLa cells infected with *C. trachomatis* (MOI 1) were incubated with either DMSO or 5 µM iAkt from 2h p.i. until fixation at 48h p.i. Infected cells were cultured without/with penicillin. *C. trachomatis* were stained with FITC-labeled anti-chlamydia MOMP (green). Inclusion area was measured by immunofluorescence microscopy. Scale bar = 50μm. **(F)** Quantification of chlamydial inclusion demonstrated in **(D)**. **(G)** Infected HeLa cells were lysed at 48 h p.i. The infectious particles (EBs) released were titrated on fresh HeLa cells by counting the Inclusion Forming Units (IFUs) 48 h later as described in *Material and Methods*. **(H)** The total RNA was extracted from the infected cells at 48h p.i. and then the expression of 16s rRNA gene was tested by qPCR. **(B–D, F–H)** Data represent the mean± SD of two independent experiments. **(B, F)** Kruskal-Wallis followed by Dunn’s multiple comparisons test, and **(C, D, G, H)** one-way ANOVA with Bonferroni’s multiple comparisons were used for statistical analysis. ***P < 0.001, ****P < 0.0001, ns, not significant. *P < 0.05, **P < 0.01.

HeLa cells were pretreated with either DMSO or 4μg/mL SC79 for 30 minutes, and then infected with *C. trachomatis* (MOI 1) and cultured for 48h without/with penicillin. The inclusion size was enlarged in cells with SC79 treatment, for both infection states (**Figures 3A, B**). The infectivity of bacterial progeny, and the expression of the chlamydial 16s rRNA gene in SC79-pretreated cells with acute infection was increased, whereas that in cells with the same treatment in persistent infection was not changed (**Figures 3C, D**). These data indicate that activation of Akt phosphorylation could promote the growth of inclusions even under the suppression by penicillin, but not the developmental cycle.

A previous report demonstrated a reduction of bacterial internalization caused by Akt inhibition (Capmany et al., 2019). Thus, we added iAkt at 2 h p.i. to avoid interference with internalization. HeLa cells were infected with *C. trachomatis* (MOI 1) and then exposed to either DMSO or 5μM iAkt and cultured for 48h without/with penicillin. We observed that the inclusions became smaller in cells exposed to iAkt in both two infection states (**Figures 3E, F**). The infectivity and the expression of the chlamydial 16s rRNA gene decreased significantly with either iAkt treatment or penicillin alone, or iAkt + penicillin treatment (**Figures 3G, H**). These findings indicate that iAkt had an effect similar to that of penicillin, and the combination of the two treatments exerted synergistic repressive effects in chlamydial infection.

### Rab14 Favors Acute Chlamydial Infection Rather Than Persistent Infection

**Figure 4:**
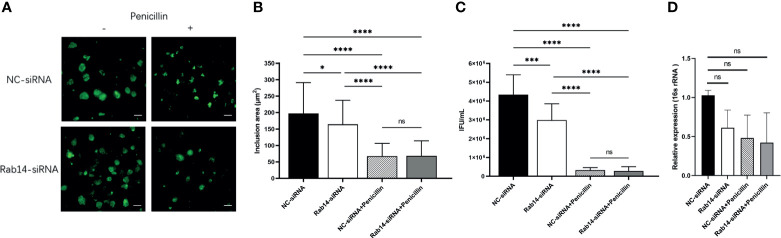
Rab14 favors acute chlamydial infection rather than persistent infection. **(A)** HeLa cells transfected with 100nM Rab14-targeting siRNA were infected with *C. trachomatis* serovar D (MOI 1) and then cultured without/with penicillin. Negative control siRNA-transfected cells were used as control. Infected cells were fixed at 48h p.i., and *C. trachomatis* were stained with FITC-labeled anti-chlamydia MOMP (green). Inclusion area was measured by immunofluorescence microscopy. Scale bar = 50μm. **(B)** Quantification of chlamydial inclusion demonstrated in **(A)**. Data represent the mean± SD. **(C)** Infected HeLa cells transfected with siRNA targeted to siRNA and NC-siRNA were lysed at 48 h p.i. The infectious particles (EBs) released were titrated on fresh HeLa cells by counting the Inclusion Forming Units (IFU) 48 h later as described in *Material and Methods*. **(D)** The total RNA was extracted from the infected cells at 48h p.i. and then the expression of 16s rRNA gene was tested by qPCR. **(B–D)** Data represent the mean± SD of two independent experiments. **(B)** Kruskal-Wallis followed by Dunn’s multiple comparisons test, and **(C, D)** one-way ANOVA with Bonferroni’s multiple comparisons were used for statistical analysis. *P < 0.05, ***P < 0.001, ****P < 0.0001, ns, not significant.

To determine the effect of Rab14 in acute and persistent chlamydial infection, we knocked down the expression of Rab14 in the HeLa cells using short interfering RNA (siRNA) transfection. HeLa cells were transfected with 100nM of chemically synthesized siRNA targeting Rab14, or a negative control siRNA. We used immunoblotting to confirm the efficacy of gene knockdown to Rab14 (reaching 70%) (**Figure S4A**), and used the CCK-8 assay to confirm the non-toxicity of siRNA to the HeLa cells (**Figure S4B**). At 72h post-transfection, cells were infected with *C. trachomatis* and cultured for 48h without/with penicillin. The cells were fixed and the chlamydial inclusions were detected using a goat polyclonal antibody targeting *C. trachomatis* MOMP coupled to FITC. In acutely infected cells, knockdown of Rab14 reduced the enlargement of chlamydial inclusions. In contrast, no changes occurred in the persistently infected cells, suggesting that Rab14 had a limited effect on the development of the inclusion in persistent infection (**Figures 4A, B**). We next collected the infectious particles from the cells and then titrated them on fresh HeLa cells by counting IFUs. The infectivity of bacterial progeny was reduced in acutely infected cells after Rab14-silencing. Nevertheless, knockdown of Rab14 did not change the bacterial infectivity, or the expression of the chlamydial 16s rRNA gene in persistent infection (**Figures 4C, D**). Taken together, these results indicate that Rab14 exerted limited effects on persistent chlamydial infection. Therefore, we suspect that the effects of Akt phosphorylation on *Chlamydia* development may not be mediated by Rab14 in persistent chlamydial infection.

### *Chlamydia*-Induced Golgi Fragmentation Is Influenced by Akt Phosphorylation

**Figure 5:**
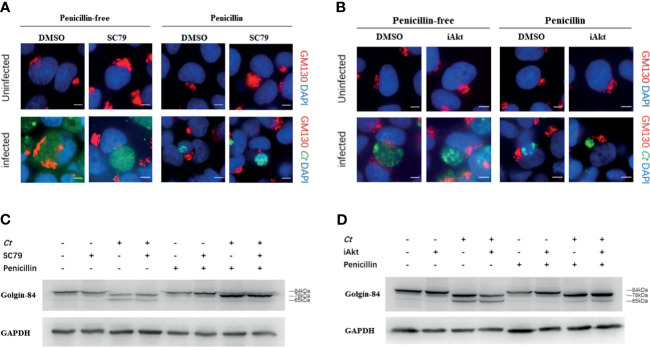
Chlamydia-induced Golgi fragmentation is influenced by Akt phosphorylation level. **(A)** HeLa cells were pretreated with either DMSO or 4μg/mL SC79 for 30min and then infected with *C. trachomatis* (MOI 1). **(B)** HeLa cells infected with *C. trachomatis* (MOI 1) were incubated with either DMSO or 5 µM iAkt from 2h p.i. until fixation at 48h p.i. **(A, B)** Uninfected cells were used as control. Cells were cultured without/with penicillin and fixed at 48h p.i. *C. trachomatis* were stained with FITC-labeled anti-chlamydia MOMP (green). The GM130 was stained with rabbit monoclonal anti-GM130 antibody labeled with Cy3 (red). Bacterial and eukaryotic DNA was labeled with DAPI (blue). Scale bar = 20μm. Images are representative of two independent experiments. **(C)** Golgin-84 cleavage in infected and uninfected cells with SC79 treatment. **(D)** Golgin-84 cleavage in infected and uninfected cells with iAkt treatment. **(C, D)** Cells were lysed in RIPA buffer with protease and phosphatase inhibitors at 48h p.i. and proteins were separated by SDS-PAGE. Proteins were transferred to PVDF membranes followed by immunoblotting with antibodies specific for golgin-84. GAPDH was used as loading control. The results are representative of three independent experiments.

We have previously discovered that persistent chlamydial infection causes little Golgi fragmentation (Zhu et al., 2014). Our current results showed that Akt phosphorylation has an impact on persistent chlamydial infection, and we wondered whether alteration of Akt phosphorylation level interferes with *Chlamydia*-induced Golgi fragmentation. We first examined Golgi morphology using immunofluorescence. Exposure to SC79, iAkt, or penicillin did not change Golgi apparatus structure in uninfected cells. The Golgi element showed a strip-like dispersed distribution surrounding inclusions in acutely infected cells, and this phenomenon was further decreased with SC79 pretreatment. In persistently infected cells, the Golgi apparatus is closely associated with the inclusions and the cell nucleus, and have a compact structure, but were observed in low amounts in SC79-pretreated cells (**Figure 5A**). However, when acutely infected cells were treated with iAkt, the Golgi apparatus remained a condensed structure similar to the persistently infected cells. Moreover, the combination of iAkt and penicillin caused an obvious reduction in chlamydial inclusion size with little Golgi fragmentation (**Figure 5B**).

We next assessed the amount and size of golgin-84, a Golgi matrix protein, in infected and uninfected cells treated with either SC79 or iAkt by immunoblotting. Golgin-84 was processed into two distinct fragments (78 kDa and 65 kDa) in the lysates of acutely infected cells, but the smaller fragment could hardly be seen in persistently infected cells. Strikingly, golgin-84 could be further processed in the infected cells with SC79 treatment but less in cells inhibited by iAkt (**Figures 5C, D**).

Taken together, our results suggests that *Chlamydia*-induced Golgi fragmentation could be regulated by Akt phosphorylation and further strengthens the hypothesis that Golgi fragmentation can influence *C. trachomatis* infections.

## Discussion

In our study, we demonstrated that Akt phosphorylation might regulate *Chlamydia*-induced Golgi fragmentation but not *via* Rab14, thereby influencing the development of *C. trachomatis* in persistent infection. Based on our results, we speculate that Akt may represent a potential supplementary therapeutic target, in combination with antibiotics for the treatment of persistent infection.

In persistent infection, *Chlamydia* slows down DNA replication and continues to transcribe genes, but stops dividing, becoming viable but non-cultivable (Muramatsu et al., 2016). Penicillin, a factor that induces persistent chlamydial infection, exerts its effect by blocking the cross-linking of peptidoglycan, a key step in cell wall synthesis in bacteria. Penicillin does not prevent inclusion formation, but can reduce infectious progeny by 95% (Kintner et al., 2014). Amoxicillin-induced persistence in mice infected intra-vaginally with *C. muridarum* resulted in increased failure of subsequent treatment with azithromycin (Kintner et al., 2014). These results might explain the clinical condition observed in patients asymptomatically infected with *Chlamydia*, who were prescribed beta-lactams due to other infections, leading to a persistent infection. These patients may confer increased resistance to other antibiotics including first-line drugs.

As *Chlamydia* acquires nutrients from the host cells (Saka and Valdivia, 2010; Mehlitz et al., 2017), Rabs, which act as the important regulators of vesicle transport (Stenmark, 2009), take part in this process. A variety of Rabs have been proved to be located on the inclusion membrane, indicating that *Chlamydia* can interact with Rabs to intercept vesicle transport (Rzomp et al., 2003; Rzomp et al., 2006; Rejman Lipinski et al., 2009). AS160 is a downstream effector of the PI3K/Akt pathway and acts as a GTPase Activating Protein (GAP) for several Rabs (Miinea et al., 2005; Capmany et al., 2019) while its phosphorylation results in the loss of GAP activity. Akt phosphorylation triggers the inactivation of AS160, and consequently slows GTP hydrolysis, prolonging the active duration of Rabs.

Rab14 controls the transport of Golgi-endosomes and the TGN-plasma membrane (Proikas-Cezanne et al., 2006). Previous studies have found that *C. trachomatis* serovar L2 recruits Rab14, which promotes the transport of sphingomyelin to the chlamydial inclusions (Capmany and Damiani, 2010). In our study, by comparing the distribution pattern of Rab14 in both acutely and persistently infected cells, we found that Rab14 recruitment to inclusions occurs in the later stages of persistent infection. In persistent infection, *Chlamydia* shows reduced metabolism, resulting in less utilization of nutrients, including sphingolipids, transported by Rab14-positive vesicles. This may explain the delayed recruitment of Rab14 to the inclusions in persistently infected cells. However, further experiments are needed to delimit the border of inclusions as the infection was processed.

We investigated Akt phosphorylation in both acutely and persistently infected cells by immunoblotting; however, our results were inconsistent with those of a previous report (Capmany et al., 2019). The differences in phosphorylation level could be due to different *Chlamydia* species or serovars, experimental conditions or host cell type. Unlike the previous study, we focused on persistent infection and discovered a lower level of Akt phosphorylation in persistently infected cells. Our results showed that *Chlamydia* growth and the infectivity of progeny can be affected by changes in Akt phosphorylation. SC79 can counteract the penicillin-induced inhibitory effect, denoted by a larger inclusion area while iAkt shows a synergistic suppression effect with penicillin. Due to the strong suppression effect of penicillin, no significant change in infectivity or the expression of chlamydial 16s rRNA gene was observed. However, we found that Rab14 knockdown exerted a limited effect on persistent infection. Taken together, Rab14 may not be the main downstream effector of Akt phosphorylation in persistent chlamydial infections. As AS160 can also act as a GAP for several Rabs, other members of the Rab family may be involved in persistent infection.

Akt inhibitors have been shown to impede Golgi-derived sphingolipid transport in acutely infected cells (Capmany et al., 2019). Our previous research demonstrated that persistent chlamydial infection reduces Golgi fragmentation (Zhu et al., 2014). The cleavage of Golgin-84 could cause Golgi fragmentation to increase sphingolipid transport towards chlamydial inclusions (Heuer et al., 2009). In our study, we observed that Golgi elements were further dispersed in infected cells with SC79-treatment, while iAkt treatment halted this process. Akt phosphorylation could regulate Golgi-associated Rab14, while Rab6 and Rab11 were shown to regulate Golgin-84-dependent Golgi fragmentation in *Chlamydia*-infected cells (Rejman Lipinski et al., 2009). We found that golgin-84 could be further processed in the infected cells with SC79 treatment, but less so in cells inhibited by iAkt, in the two types of infection states evaluated, suggesting that Akt phosphorylation affects the *Chlamydia*-induced Golgi fragmentation.

Our data showed that in persistent infection, the Akt inhibitor could impede *Chlamydia* development and *Chlamydia*-induced Golgi fragmentation, whereas Rab14 exerted limited effects. In addition, the PI3K/Akt signaling pathway is involved in chlamydial infection, including invasion (Lane et al., 2008; Patel et al., 2014; Subbarayal et al., 2015), resistance to apoptosis (Rajalingam et al., 2008; Zou et al., 2018), and stress response (Thapa et al., 2020). Therefore, Akt appears to be a more valuable upstream therapeutic target than Rab14 in persistent chlamydial infection. A recent study has demonstrated that several inhibitors of the ERK/RSK pathway represent potential treatment options for Host-directed therapy (HDT), limiting the development of *Chlamydia* (Xue et al., 2020). Our study suggests that Akt inhibitors could also be a member of HDT, and help treat both acute and persistent chlamydial infection.

In summary, we have provided evidence that Rab14 recruitment and Akt phosphorylation differ between persistent and acute chlamydial infections. We found that in persistent infection, Rab14-silencing showed limited influence, whereas Akt phosphorylation exerted greater effects on inclusion development than infectivity. We have also demonstrated that Akt phosphorylation regulates *Chlamydia*-induced Golgi fragmentation. Therefore, we suspected that in persistent chlamydial infection, Akt phosphorylation may influence *Chlamydia* development and regulate Golgi fragmentation without involving Rab14. Our study also provides new insight into the potential for synergistic repressive effects of Akt inhibitors and antibiotics in the treatment of persistent chlamydial infection induced by penicillin.

## Data Availability Statement

The raw data supporting the conclusions of this article will be made available by the authors, without undue reservation.

## Author Contributions

XH and JT were the main investigators of the study and drafted the manuscript. ML and WL contributed in performing the experiment and analyzing the data. XC, HZ and ZH contributed to the conception and design of the work. JH and CM were the corresponding authors who contributed in designing the research project, funding, and revising the manuscript. All authors contributed to the article and approved the submitted version.

## Funding

This study was supported by Science and Technology Projects in Guangzhou, China (grant number 201807010081) and the Guangdong Natural Sciences Foundation of China (grant number 2018A030310270).

## Conflict of Interest

The authors declare that the research was conducted in the absence of any commercial or financial relationships that could be construed as a potential conflict of interest.
