# Supplementary material for: Akt Phosphorylation Influences Persistent Chlamydial Infection and Chlamydia-Induced Golgi Fragmentation Without Involving Rab14
Source: Front Cell Infect Microbiol. 2021 Jun 8;11:675890. doi: 10.3389/fcimb.2021.675890 (PMC8218875; doi:10.3389/fcimb.2021.675890)
Supplement: Supplementary file 1 [file DataSheet_1.pdf]

A

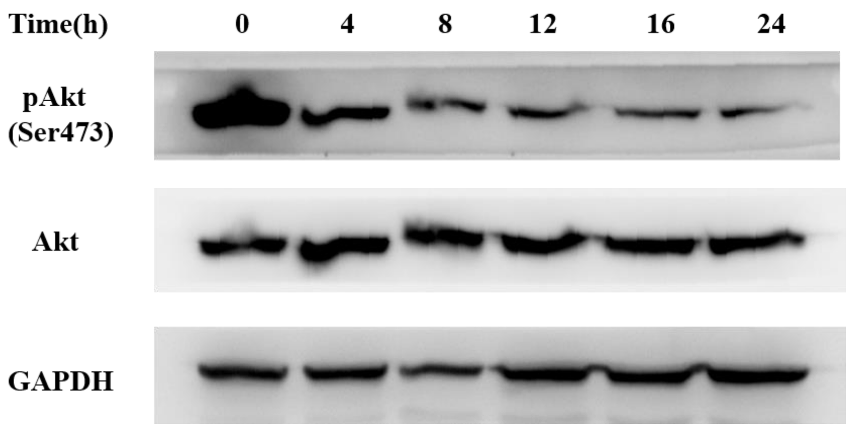

B

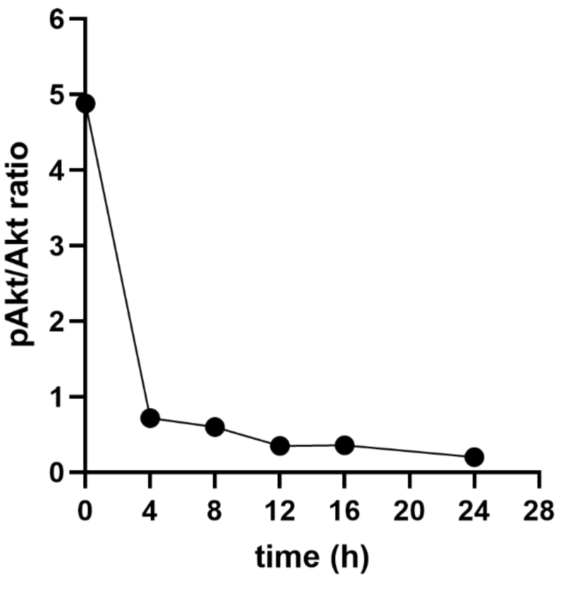

Figure S1. Akt phosphorylation of Hela cells with FBS-starved treatment for different times. (A) Immunoblotting analysis of phosphorylated Akt (pAkt) and total Akt in the lysates of Hela cells. Akt and phosphorylated Akt expression were evaluated by immunoblotting with anti-Akt and anti-pAkt antibodies, respectively. GAPDH was used as loading control. **(B)** Phosphorylation ratio of pAkt/Akt demonstrated in (A).

A

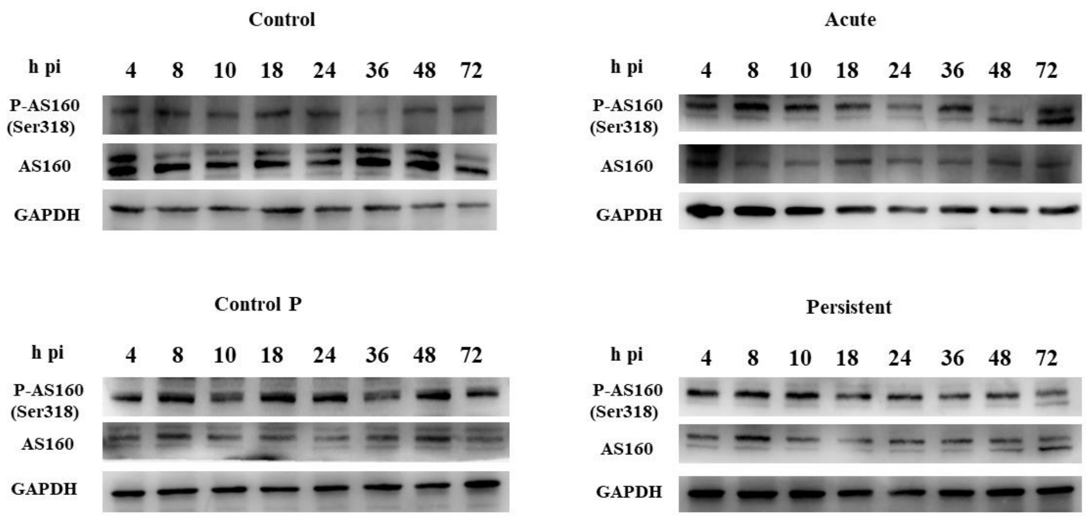

B

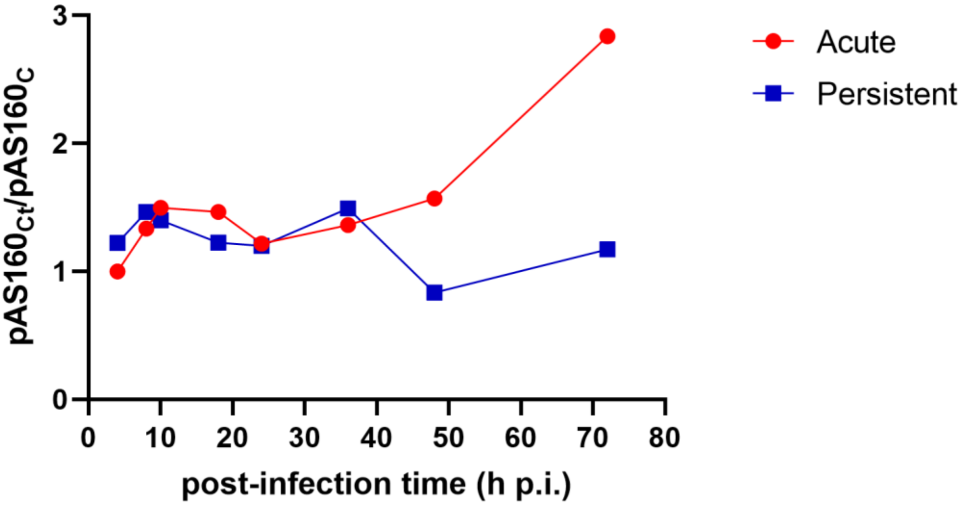

Figure S2. Different AS160 phosphorylation level in acute and persistent chlamydial infection. (A) HeLa cells were infected with *C. trachomatis* (MOI 1) and cultured without/with penicillin. The lysates were harvested at the indicated periods of time. Uninfected cells treated without/with penicillin were used as control (Control and Control P). Cells were lysed in RIPA buffer with protease and phosphatase inhibitors and proteins were separated by SDS-PAGE. Proteins were transferred to PVDF membranes followed by immunoblotting with antibodies specific for Akt and phosphorylated AS160. GAPDH was used as loading control. Cells were FBS starved 4 h before sampling. The results are representative of two independent experiments. **(B)** Relative phosphorylation level of AS160 in chlamydial infected cells compared to pAS160 in uninfected cells.

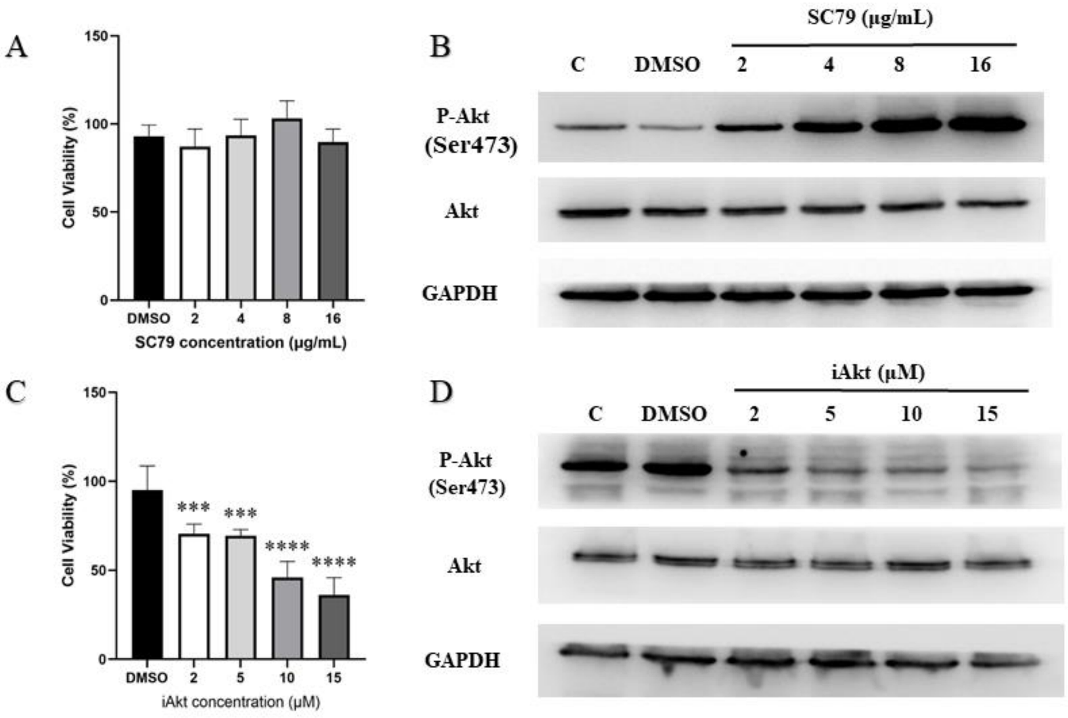

Figure S3. Results of CCk-8 assay and immunoblotting. (A) Hela cells were pretreated with either DMSO or different concentration of SC79 for 30 min, followed by culture for 48h. **(B)** Hela cells were treated with either DMSO or different concentration of iAkt for 48h. **(A, B)** Cell viability was determined by Cell Counting Kit (CCK)-8 assay. Data represent the mean ± SD. One-way ANOVA with Bonferroni’ s multiple comparisons were used for statistical analysis. *** P<0.001， **** P<0.0001.**(C)** Hela cells were pretreated with either DMSO or different concentration of SC79 for 30 min, followed by culture for 48h. **(D)** Hela cells were cultured for 48h, treated with DMSO and different concentration of iAkt 4 h before harvesting. **(C,D)** Akt and phosphorylated Akt (pAkt) expression were evaluated by immunoblotting with anti-Akt and anti-pAkt antibodies, respectively. GAPDH was used as loading control. Cells were FBS starved 4 h before sampling. **(A, B, C, D)** Blank cells were used as control (C).

A

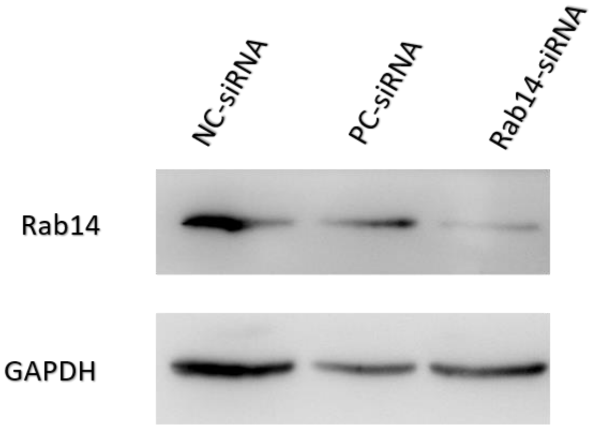

B

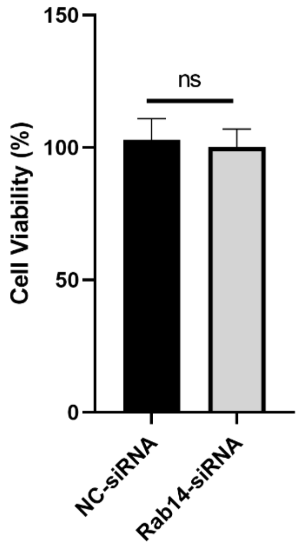

Figure S4. The knockdown efficacy of siRNA and cell viability of siRNA-treated cells. Hela cells were transfected with 100nM negative control siRNA (NC), positive control siRNA (PC, targeted to GAPDH), and siRNA targeted to Rab14 for 72h. **(A)** Immunoblotting analysis of Rab14 in the lysates of siRNA-treated cells. GAPDH was used as loading control. **(B)** CCK-8 assay were used to evaluate the cell toxicity of siRNA. Data represent the mean±SD. Unpaired t test was used for statistical analysis. ns, not significant.
